# Prevalence and Antimicrobial Susceptibility Pattern of* E. coli* O157:H7 Isolated from Traditionally Marketed Raw Cow Milk in and around Asosa Town, Western Ethiopia

**DOI:** 10.1155/2017/7581531

**Published:** 2017-02-20

**Authors:** Nigatu Disassa, Berhanu Sibhat, Shimelis Mengistu, Yimer Muktar, Dinaol Belina

**Affiliations:** ^1^Assosa University, P.O. Box 18, Assosa, Ethiopia; ^2^Haramaya University College of Veterinary Medicine, P.O. Box 138, Dire Dawa, Ethiopia

## Abstract

A cross-sectional study was conducted from October 2014 to July 2015 to determine the prevalence and populations of* E. coli* as well as the prevalence and antimicrobial susceptibility of* E. coli* O157:H7 isolated from raw milk. Biochemical and serological tests methods were used to confirm* E. coli* and* E. coli* O157:H7 and isolates were subjected to antimicrobial susceptibility test using the agar disc diffusion method. Out of 380 raw milk samples examined, 129 (33.9%) and 11 (2.9%) were contaminated with* E. coli* and* E. coli* O157:H7, respectively. The highest prevalence was recorded in samples obtained from vendors (39.1%, 4.978 ± 0.180 log_10_/ml) compared with samples from farmers (28.1%, 3.93 ± 0.01 log_10_/ml) with significant differences (*P* = 0.02). The frequency of contamination was higher in the samples collected from milk that was stored and transported in plastic containers (39.4%) than in the containers made of stainless steel (23.0%) (*P* = 0.002). The antimicrobial susceptibility profile showed that* E. coli* O157:H7 were resistant to tetracycline (81.8%), streptomycin (81.8%), and kanamycin (63.6%). Milk samples were produced and handled under poor hygienic conditions, stored, and transported in inappropriate containers and under temperature abuse conditions leading to high health risk to the consumers. Additional studies would be needed to establish association between the occurrences of* E. coli* O157:H7 in raw milk and all the risk factors involved in and around Asosa town.

## 1. Introduction

*Escherichia coli* is a normal inhabitant of the intestines of animals and humans; its recovery from food may be of public health concern due to the possible presence of enteropathogenic and/or toxigenic strains which lead to sever gastrointestinal disturbance. It is among many pathogenic microorganisms which can get access to milk and dairy products and is considered as a reliable indicator of contamination by manure, soil, and contaminated water [1, 2].

Outbreaks of VTEC infections involving serogroup O157 have been reported from different countries of the world including United States, Canada, Asia, Australia, Europe, and Africa through various sources of infection and different case fatality [3]. In southern Africa and Swaziland in 1992 an outbreak of* E. coli* O157:H7 affecting thousands was attributed to contamination of surface water with cattle dung and animal carcasses. Dairy products (milk and cheese), both unpasteurized and pasteurized, of bovine and ovine origin have been implicated in VTEC infections. This has included a number of outbreaks among children that have been attributed to the consumption of raw milk and dairy products. Antibiotic use in VTEC infections is controversial because of the potential to increase production and secretion of Shiga toxins. However, increase in antibiotic resistance has been noted over the last 20 years [4–7].

Food borne diseases are common in developing countries, including Ethiopia, because of the prevailing poor food handling and sanitation practices, inadequate food safety laws, weak regulatory systems, lack of financial resources to invest in safer equipment, and lack of education for food-handlers. The National Hygiene and Sanitation Strategy program reported that about 60% of the disease burden was related to poor hygiene and sanitation in Ethiopia. Unsafe sources, contaminated raw food items, improper food storage, poor personal hygiene during food preparation, inadequate cooling and reheating of food items, and a prolonged time lapse between preparing and consuming food items have been identified as contributing factors for outbreaks of food borne diseases [8, 9].

The consumption of raw milk and its derivatives is common in Ethiopia, which is not safe from a consumer health point of view as it may lead to the transmission of various diseases [10]. The ability of raw or processed milk to support the growth of several spoilage or pathogenic microorganisms can lead to spoilage of the product or infections and intoxications in consumers. The most commonly known bacterial pathogens still of concern today in raw milk and other dairy products include* Bacillus cereus*,* Listeria monocytogenes*,* Yersinia enterocolitica*,* Salmonella* spp.,* Escherichia coli*, and* Campylobacter jejuni*, among which* E. coli* O157:H7 was the focus of this study [1, 11, 12].

Even though milk represents an important food in consumers' nutrition as well as in the nutrition and income of producers, there is limited work so far undertaken regarding the assessment of the bacteriological quality and safety of raw cow milk in western Ethiopia, in general, and in Assosa town, in particular. Therefore, the aim of the present study was to determine the prevalence and antimicrobial susceptibility pattern of* E. coli* and* E. coli* O157:H7 and to evaluate the hygienic condition of raw cow milk at two different points that are considered as critical points (farmers and vendors) in the value chain in Asosa of western Ethiopia.

## 2. Materials and Methods

### 2.1. Description of the Study Area

Asosa is a town found in Asosa zone in western Ethiopia and the capital of the Benishangul-Gumuz Regional State, Ethiopia. The town is located at latitude and longitude of 10°04′N 34°31′E, with an elevation of 1570 meters, at 476 km distance from the capital city of the country, Addis Ababa. In this area, the mixed farming system is dominant, in which about 92.5% of the population is engaged in agriculture as a major means of subsistence. The region is bordered with the Sudan in the west, Amhara Regional State in the east and north, Oromia Regional State in the east and south east, and Gambella Regional State in the south. Three administrative zones, one special woreda, 19 woredas, and 425 kebeles exist in the region. It covers a total area of about 5,038,100 hectares. Plain undulating slopes and mountains characterize the topography of the region. The agroclimatic zone of the region is categorized as 75% “*kola*,” 24% “*woinadega*,” and 1% “*dega.*”

The rainfall distribution pattern is monomodal, commencing towards the end of April and ending in October. The topography of the woreda is mainly plain [13].

### 2.2. Study Design

A cross-sectional study was conducted from October 2014 to July 2015 to determine the prevalence and populations of* E. coli* with emphasis on* E. coli* O157:H7 in bovine milk and to determine the antimicrobial sensitivity of the* E. coli* O157:H7 isolates.

### 2.3. Sample Size Determination

The prevalence of the selected bacteria at the study area was not known; hence, the required sample size was calculated considering a previously published prevalence estimate of 44.4% [10] reported from a study on the microbial quality of milk in Mekelle town. Accordingly, a total of 380 (178 from farmers and 202 from venders) raw cow milk samples, each consisting of 25 ml, were collected at weekly intervals.

### 2.4. Sampling Methods and Procedures

First, a baseline survey was conducted to identify the total number of farms, farm size, farming system, and the status and number of vendors in and around the Asosa town. According to the results of the survey all farms were at the household/smallholder level, with farm size not more than 4 cows per farm. Then, a total of 60 small farms and 25 small vendors were identified as main sources of milk for consumers in and around the town and included in the study using stratified sampling methods. A simple random sampling technique was applied to collect raw milk samples from each group of vendors. Then all individuals involved directly or indirectly in milk production and marketing were communicated to allow us permission to obtain samples and provide us with relevant information. After that, 178 milk samples were collected aseptically using sterile test tubes from milking buckets immediately after milking and 202 raw milk samples were collected from vendors' containers. The samples were transported to the Asosa Regional Veterinary Research Laboratory under refrigeration (using ice boxes) for bacteriological analysis. The examination of milk samples was conducted within 4 hours after collection. Isolation and identification of* E. coli* O157:H7 were also conducted in the laboratory and antimicrobial sensitivity test was undertaken for* E. coli* O157:H7 isolates.

In addition to this, questionnaires and observation check lists were used as a tool to gather information (data) on the hygienic practices during milking, handling, storage, transportation, duration of transportation, and storage of the milk by the stakeholders and their knowledge regarding diseases associated with milk, in order to assess the associated risks.

## 3. Study Methodologies

### 3.1. Isolation and Identification of* E. coli* and* E. coli* O157:H7

Each raw cow milk sample was enriched using EC-broth at 37°C for 24 hours, inoculated on MacConkey agar, and then incubated at 37°C for 24 hours. Typical colonies on MacConkey agar (pink, due to their ability to ferment lactose) were stained using gram stain and observed for their staining and morphological characteristics and transferred to eosin-methylene-blue (EMB) agar. The colonies with metallic sheen on EMB agar which is typical feature of* E. coli* were transferred to sorbitol MacConkey agar to check the presence of* E. coli* O157:H7 phenotype (inability to ferment sorbitol). Then the confirmed pure cultures considered as* E. coli* positive were transferred to nutrient agar to be used for additional confirmatory biochemical tests (IMViC tests) and serological tests as described below [14]

### 3.2. Serological Test

The* E. coli-*sorbitol-negative colonies were serologically confirmed by using* E. coli* O157:H7 latex agglutination assay containing latex particles coated with antibodies specific for the* E. coli* O157 and the* E. coli* H7 antigen. Identification of* E. coli* O157:H7 was carried out following the manufacturer's instructions; hence colonies that agglutinated were considered to be* E. coli* O157:H7.

### 3.3. Enumeration of* E. coli*

Milk samples (25 ml) were diluted in buffered peptone saline water (225 ml); serial dilution of 10^−1^, 10^−2^, and 10^−3^ was applied in order to quantify this microbial group. Most probable number (MPN) method was used after serial dilutions to estimate the populations of* E. coli*.* E. coli* was enumerated by growing each serial dilution in 3 test tubes in* Escherichia coli* broth (EC-broth) after 24/48 hours of incubation at 45°C as stated in [14].

### 3.4. Antimicrobial Susceptibility Testing

Mueller-Hinton agar media were used for susceptibility testing according to the criteria of the National Committee for Clinical Laboratory Standards [15]. The isolated* E. coli* O157:H7 strains were tested for sensitivity to the most commonly used antimicrobials including, cefoxitin (CF) (5 *μ*g), gentamicin (GCN) (10 *μ*g), kanamycin (K) (30 *μ*g), norfloxacin (NOR) (10 *μ*g), streptomycin (S) (10 *μ*g), trimethoprim-sulfamethoxazole (SXT) (25 *μ*g), and tetracycline (TE) (30 *μ*g).

### 3.5. Data Analysis

The data obtained was coded and entered in Excel 2010 for storage and then entered in to data editor view of SPSS (Version 20) for statistical analysis. Cross tabulation was used to calculate the frequencies of the parameters of the variables. In some cases, the chi-square statistic was used to test for significant difference in prevalence of* E. coli* in raw milk samples collected from raw milks managed in different conditions considered as risk factors. The level of significance was set at 0.05. The microbiological count data per milliliter of sample was transformed to logarithm of base ten (log counts ml^−1^) before statistical analysis and the results are presented as the geometric means.

## 4. Results and Discussion

### 4.1. Microbiological Isolation and Identification

Out of the 380 raw cow milk samples collected in the present study,* E. coli* was isolated from 129 (33.9%) of the samples based on morphological and cultural characteristics and also biochemical tests (Table 1). This prevalence is lower when compared to reports of [10] from Mekelle town, [16] from Britain, [17] from South India, and [18] from Malaysia who reported prevalence of* E. coli* from raw milk of 44.4%, 63%, 70%, and 65%, respectively. On the other hand, [19] reported a 38.0% prevalence from India

The variation that was seen in prevalence in different studies may be due to difference in sample size, farming system, farm size, milking equipment, milking technique, geography, ecology, duration of milk transportation, and hygienic conditions [20]. The presence of* E. coli* may not necessarily indicate a direct fecal contamination of milk but is an indicator of poor hygiene and unsanitary practices during milking and further handling of milk and presents a potential hazard for people consuming such products [21].

The frequency of contamination of* E. coli* was significantly higher (*P* = 0.02) in raw milk samples collected from vendors (39.1%) than from different farms (28.1%) and also significantly increased (*P* = 0.001) as the time required for the milk to reach the market increased. The container in which milk was collected was evaluated and a higher rate of contamination was detected in the samples collected from milk held in plastic containers than stainless steel. The observed differences are probably due to the longer time taken for the milk to reach the market at ambient temperature under poor hygienic conditions which support the growth of the bacteria in the milk samples taken from the vendors.

In this study, the methods of production, transportation, handling, and sale of milk are prone to contamination. Hence, milk can be easily contaminated from different sources including the contaminated udder, milk handlers with poor personal hygiene, water of poor quality, and inappropriately cleaned and/or sanitized containers, all of which contribute to milk contamination [16, 18, 21].

In the current study, the overall prevalence of* E. coli* O157:H7 was found to be 2.9% (11/380). According to the results indicated in Table 1, a greater prevalence was observed in the milk samples collected from milk venders (5.0%) compared to those collected from the farmers (0.6%) and also in milk samples collected in containers managed under poor hygienic condition (4.3%) than from properly cleaned containers (0.7%) (Table 1). In both cases, the observed differences were statistically significant (*P* < 0.05). The containers made of plastic were identified to be more prone to be contaminated by* E. coli* O157:H7 than stainless steel, but the difference was not statistically significant. There are a number of studies from different countries of the world concerning the prevalence of* E. coli* O157 in raw milk. Arafa and Soliman [21] reported that of raw milk and fresh cream examined in Egypt 2.6% and 1% were contaminated with* E. coli* O157:H7, respectively. Allerberger et al. [22] reported 3% of the milk samples tested in Austria to be positive for* E. coli* O157:H7, Klie et al. [23] found that 3.9% of the raw milk analyzed in Germany was contaminated with* E. coli* O157:H7, and Chye et al. [18] detected* E. coli* O157:H7 in 33.5% of raw milk samples in Malaysia. This might be due to differences in animal management, milking system, and milk handling practices among different regions.

### 4.2. Enumeration of* E. coli*

Most probable number (MPN) is one of the most commonly used methods to estimate the microbial load from milk and milk products. The mean MPN value of* E. coli* in raw milk samples was calculated with respect to different conditions that were considered as risk factors. Slightly higher mean MPN values (log_10_) were obtained in samples collected from vendors (4.978 ± 0.180/ml) than in milk collected from farmers (4.720 ± 0.203/ml), whereas milk held in plastic containers had a higher mean* E. coli* count (4.926 ± 0.157/ml) than the milk held in stainless steel (4.710 ± 0.264/ml). Similarly, samples collected from milk managed under poor hygienic conditions had a higher mean count (5.016 ± 0.151/ml) than the samples collected from milk managed under good hygienic condition (4.556 ± 0.278/ml). At the same time the load of* E. coli* increased with time, with the highest mean value (6.161 ± 0.478/ml) detected in the samples collected from the milk that had taken 4:01–5 hours to reach the market (Table 2).

According to an earlier report, the* E. coli* count in milk samples obtained from vendors was significantly higher (3.64 ± 0.776 cfu/ml) than milk samples obtained from dairy farms (3.93 ± 0.01 cfu/ml) [24], suggesting that allowing the milk samples' temperature to stay in the environmental temperature zone will favour the growth of* E. coli* and could be responsible for the high observed counts. There are several reasons for these variations, such as differences in hygienic practices during milking, differences in geographic location, and differences in seasonal trends [21].

In the study area, milking animals were kept with the rest of the stock in a shade or enclosure during the night. Milking was done in the shaded grazing field in front of the homestead, or under trees. However, as these areas were not generally kept clean enough, cows usually become soiled with dung and urine. Moreover, cleaning of the udder and of the hind quarters of the cows was not a common practice among milkers. This, coupled with the unhygienic cleaning and handling of milk containers, resulted in microbial contamination of milk [25, 26].

The bacterial counts in milk probably reveal the general conditions of sanitation and temperature control under which milk was produced, handled, and held [21]. In earlier studies,* E. coli* was detected as the most abundantly isolated species in raw milk sampled from smallholder producers in the central highlands of Ethiopia [12, 27], which is a good indicator of fecal contamination [28].* E. coli* are often used as indicator microorganisms, and high populations of* E. coli* imply a risk that other enteric pathogens may be present in the sample [21].

Possible reasons for the high counts could be due to infected udders of the cows, use of unclean equipment, poor personal hygiene, lack of cooling after milking, and lack of heat treatment, which contribute to the poor hygienic quality of raw milk. Therefore, training and guidance in general milking hygienic practices and in keeping milk at low temperature should be given to the farmers to avoid microbial growth and lengthen the shelf life of milk [29].

The average values (ranging from 4.258 to 6.161, Table 3) in the present study indicated that the milk for consumers was of inferior quality and should be considered as unsafe. Since* E. coli* are indicator organisms for fecal pollution and* E. coli* O157:H7 often coexists with other coliform pathogens, the high enumerated levels of* E. coli* might be enough to say the milk is unsafe for consumers.

### 4.3. Antimicrobial Resistance Pattern of* E. coli* O157:H7

The susceptibility of the* E. coli* O157:H7 isolates against seven commonly used antimicrobials was tested and the isolates (*n* = 11) were characterized as susceptible, intermediate, and resistant based on the size of zone of inhibition [15]. According to the test results most of the* E. coli* O157:H7 (≥50%) isolates were resistant to tetracycline (81.8%), streptomycin (81.8%), kanamycin (63.6%), cefoxitin (54.5%), and norfloxacin (54.5%) (Table 3). Development of antibiotic resistance among bacteria such as* E. coli* poses an important public health concern. Effectiveness of treatments and ability to control infectious diseases in both animals and humans may be severely hampered [19]. The currently recommended management of an infection mainly relies on supportive therapy and hydration [30].

### 4.4. Questionnaire Data and Its Interpretation

A total of 125 respondents were included in the study to collect relevant information regarding their knowledge about keeping the quality of the milk, the hygienic status, the type of milk containers, and the elapsed time for the milk to reach the market. Respondents were categorized into 4 groups including farmers (32.0%) (40), venders (32.0%) (40), cafeteria (12.0%) (15), and household consumers (24.0%) (30).

According to the findings summarized in Table 4 most of milk supplied to consumers in the town was originated from village around the town which was transported for a longer time to reach the market and managed by people with mostly unsatisfactory knowledge on the keeping quality of milk in plastic containers. Similarly, the sources of water for sanitation were underground water wells, pipes, or both wells and pipes with poor frequency of sanitation (Table 4). All these conditions favour the introduction and multiplication of pathogenic bacteria in the milk. These bacteria will reach the consumer through consumption of improperly cooked/boiled milk and improperly treated/prepared “ergo” or cheese since milk is also consumed through both of these forms.

## 5. Conclusion

Most of the milk supplied to the consumer in the town was managed under poor hygienic conditions at ambient temperatures with poor levels of sanitation in plastic containers. Most of the stakeholders were managing the raw milk with limited awareness and knowledge on milk contamination and on the public health impact of milk-borne pathogens. The sources of* E. coli* in the raw cow milk may be from contaminated udders, contaminated water, poor sanitation practices, contaminated containers, and milk handlers themselves. Since the milk is managed at an ambient temperature, high microbial populations can be reached within short period of time. In this study, the presence and populations of* E. coli* as an indicator organisms and* E. coli* O157:H7 as pathogenic organisms in raw cow milk samples showed that the produced milk is of poor microbiological quality and of public health risk to the consumer.* E. coli* O157:H7 can cause infection as well as toxic infection at a very low infective dose like < 100 cells. In the study area, there is no standard hygienic conditions followed by producers during milk production. The hygienic conditions are different according to the production system, adapted practices, level of awareness, and availability of resources. In most of the cases under smallholder condition, the common hygienic measures taken during milk production especially during milking are limited to letting the calf to suckle for few minutes and/or washing the udder before milking. The quality of the water used for cleaning purposes (to wash the udder, milk equipment, and hands), however, is not secured. The study also indicated that the* E. coli* O157:H7 isolates were resistant to most of the antimicrobials used at the study area, which may exacerbate* E. coli* infections in the future.

## Figures and Tables

**Table 1:** Prevalence of *E. coli* in the study samples in different conditions.

	Tested samples	Presence of *E. coli*		Presence of *E. coli* O157:H7	
		Negative	Positive	Negative	Positive
Farm	178 (46.8%)	128 (71.9%)	50 (28.1%)	177 (99.4%)	1 (0.6%)
Vendor	202 (53.2%)	123 (60.9%)	79 (39.1%)	192 (95.0%)	10 (5%)
*X* ^2^		5.12		6.48	
*P* value		0.02		0.01	
Good^*∗*^	150 (39.5%)	112 (74.7%)	38 (25.3%)	149 (99.3%)	1 (0.7%)
Poor^*∗∗*^	230 (60.5%)	139 (60.4%)	91 (39.6%)	220 (95.7%)	10 (4.3%)
*X* ^2^		8.20		4.38	
*P* value		0.004		0.04	
Plastic^*∗∗∗*^	234 (66.2%)	154 (60.6%)	100 (39.4%)	245 (96.5%)	9 (3.5%)
Steel^*∗∗∗∗*^	126 (33.2%)	97 (77.0%)	29 (23.0%)	124 (98.4%)	2 (1.6%)
*X* ^2^		10.05		1.15	
*P* value		0.002		0.28	
<1 hour	197 (51.8%)	136 (69.0%)	61 (31.0%)	195 (99%)	2 (1%)
1–4 hours	96 (25.3%)	43 (44.8%)	26 (27.1%)	93 (96.9%)	3 (3.1%)
>4 hours	87 (22.9%)	45 (51.7%)	42 (48.3%)	81 (93.1%)	6 (6.9%)
*X* ^2^		13.2		4.81	
*P* value		0.001		0.09	

Total	380 (100%)	251 (66.1%)	129 (33.9%)	369 (97.1%)	11 (2.9%)

^*∗*^Milk containers are usually cleaned properly before and after milking using quality water with help of detergent and using of containers made of stainless steel.

^*∗∗*^The condition that does not fulfill or partially fulfill the first case.

^*∗∗∗*^Samples collected from raw cow milk held in containers made of plastic.

^*∗∗∗∗*^Samples collected from raw cow milk held in container made of stainless steel.

**Table 2:** Mean (±SE) count (log_10_/ml) of *E. coli* in raw cow milk from two different sources collected under different conditions.

	Range	Mean	95% CI for mean value		SE
			Lower	Upper	
Sources					
Farmer	1.10–7.00	4.720	4.312	5.127	0.203
Vendors	1.10–7.00	4.978	4.620	5.336	0.180
Hygienic condition					
Good	1.10–7.00	4.556	3.983	5.111	0.278
Poor	1.81–7.00	5.016	4.416	5.316	0.151
Containers					
Plastic	1.10–7.00	4.926	4.615	5.238	0.157
Stainless steel	1.10–6.13	4.710	4.170	5.250	0.264
Time ranges					
<1 hour	1.10–7.00	4.258	4.214	4.902	0.173
1-2 hours	5.35–7.00	4.425	3.336	5.513	0.481
2:01–4 hours	1.81–6.13	5.779	5.028	6.529	0.354
4:01–5 hours	1.81–7.00	6.161	4.103	8.218	0.478
>5 hours	1.81–7.00	5.239	4.626	5.853	0.297

**Table 3:** Antimicrobial resistance profile of *E. coli* O157:O7 isolates.

	Susceptible	Intermediate	Resistant
Streptomycin	0 (0%)	2 (18.2%)	9 (81.8%)
Trimethoprim- sulfamethoxazole	2 (18.2%)	6 (54.5%)	3 (27.3%)
Cefoxitin	1 (9.1%)	4 (36.4%)	6 (54.5%)
Kanamycin	0 (%)	4 (36.4%)	7 (63.6%)
Gentamycine	4 (36.4%)	3 (27.3%)	4 (36.4%)
Tetracycline	0 (0%)	2 (18.2%)	9 (81.8%)
Norfloxacin	1 (9.1%)	4 (36.4%)	6 (54.5%)

**Table 4:** Summary of questionnaire data.

Risk factors	Cafeteria (*n* = 15)	Household (*n* = 30)	Vender (*n* = 40)	Farmer (*n* = 40)	Total (*n* = 125)
Knowledge about keeping quality of milk					
Nonsatisfactory^*∗*^	12 (80.0%)	24 (80.0%)	37 (92.3%)	38 (95.0%)	111 (88.8%)
Satisfactory^*∗∗*^	3 (20.0%)	6 (20.0%)	3 (7.7%)	2 (5.0%)	14 (11.2%)
Water for sanitation					
Well	2 (10.0%)	0 (0%)	13 (65.0%)	5 (25.0%)	20 (16.0%)
Pipe	10 (73.4%)	13 (77.5%)	20 (30%)	14 (45%)	57 (45.6%)
Both	3 (6.20%)	17 (35.4%)	7 (14.6)	21 (43.8)	48 (38.4%)
Frequency of sanitation					
Usually	10 (67.5%)	12 (40.0%)	19 (47.5%)	16 (40.0%)	57 (45.6%)
Sometimes	5 (33.5%)	18 (60.0%)	21 (52.5%)	24 (60.0%)	68 (54.4%)
Washing equipment					
Only water	4 (26.7%)	12 (86.7%)	23 (82.5%)	24 (87.5%)	63 (50.4%)
Water & detergents	11 (17.7%)	18 (29.0%)	17 (27.4%)	16 (25.8%)	62 (49.6%)
Containers					
Plastic container	10 (11.5%)	24 (27.6%)	24 (27.6%)	29 (33.3%)	87 (70.7%)
Stainless steel	5 (13.2%)	6 (15.8%)	16 (42.1%)	11 (28.9%)	28 (29.3%)
Sources of milk					
Town	3 (5.1%)	16 (27.1%)	0 (0%)	40 (67.8%)	59 (47.2%)
Village^*∗∗∗*^	12 (18.2%)	14 (21.2%)	40 (60.6%)	0 (0%)	66 (52.8%)
Knowledge of keeping quality of milk gained					
From parents	3 (33.3%)	11 (53.3%)	15 (27.5%)	17 (57.5%)	46 (36.8%)
Observations	8 (14.0%)	11 (19.3%)	22 (38.6%)	16 (28.1%)	57 (45.6%)
Formal training	4 (13.4%)	8 (10%)	3 (12.5%)	7 (7.5%)	22 (17.6%)
Time required to reach the market					
≤1 hour	2 (4.0%)	7 (14.0%)	6 (12.0%)	35 (70.0%)	50 (40.0%)
1-2 hours	4 (16.7%)	6 (25.0%)	9 (37.5%)	5 (20.8%)	24 (19.2%)
2:01–5 hours	4 (15.4%)	14 (53.8%)	8 (30.8%)	0 (0%)	26 (20.8%)
>5 hours	5 (20.0%)	3 (12.0%)	17 (68.0%)	0 (0%)	25 (20.0%)

^*∗*^Lack of awareness and knowledge about sources and detection of contamination, public health impacts of contaminated milk, and necessary steps to control pathogens. ^*∗∗*^With awareness and knowledge about sources of contamination, public health impacts of contaminated milk and necessary steps to control pathogens. ^*∗∗∗*^Sources of the milk from where milk was transported on foot and took more than an hour.
